# Supplementary material for: Sister chromatid exchanges induced by perturbed replication can form independently of BRCA1, BRCA2 and RAD51
Source: Nat Commun. 2022 Nov 7;13:6722. doi: 10.1038/s41467-022-34519-8 (PMC9640580; doi:10.1038/s41467-022-34519-8)
Supplement: Supplementary file 1 — Supplementary Information [file 41467_2022_34519_MOESM1_ESM.pdf]

A

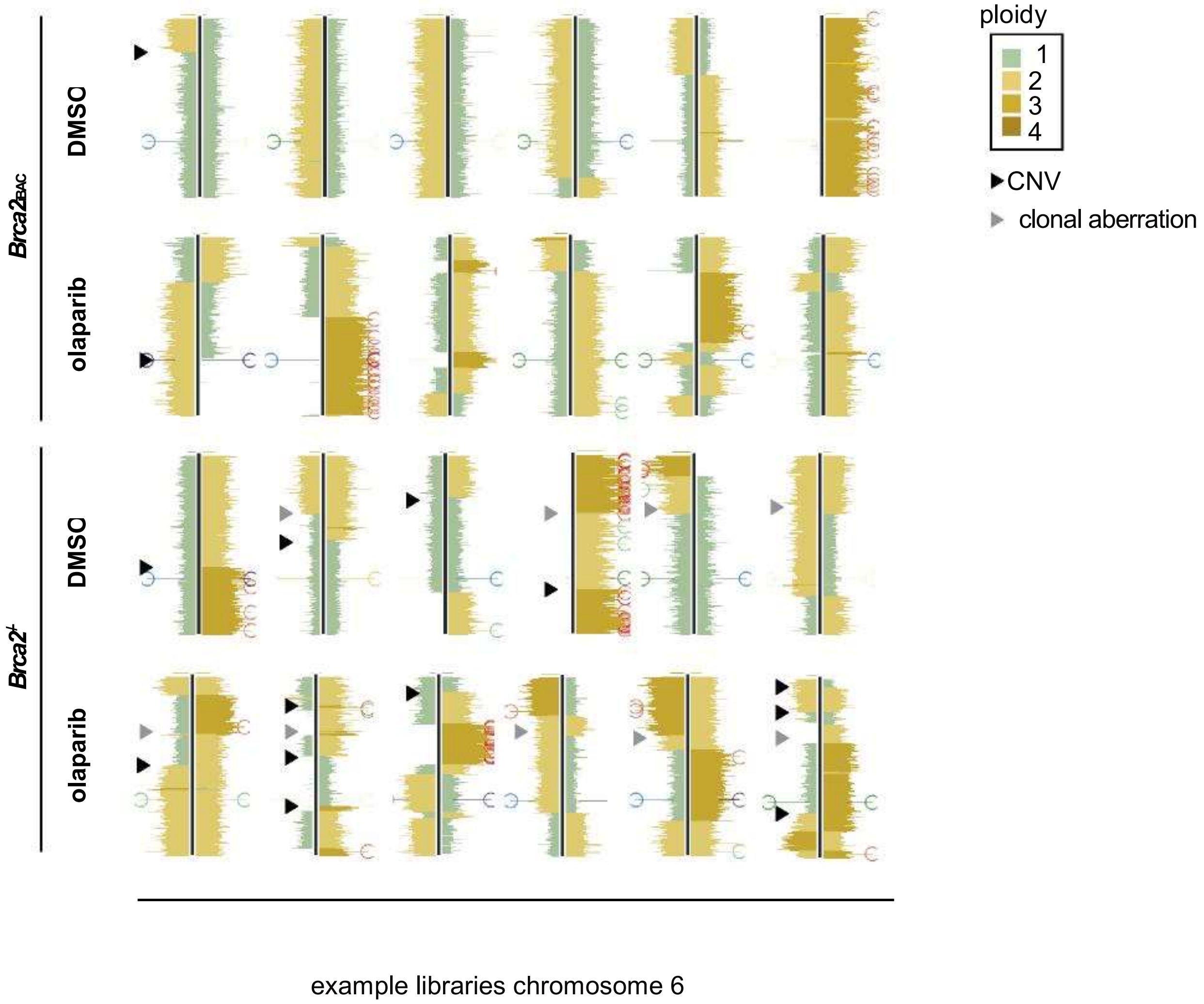

Supplementary Figure 1: Olaparib-treatment induces copy number variation in Brca2iBAC and Brca2-/- cancer cells. (A) Representative Strand-seq libraries of chromosome 6 in Brca2-/- cells or Brca2iBAC cells, treated with DMSO (top) or olaparib (bottom). Black arrowheads indicate copy number variations (CNVs), grey arrows indicate clonal aberrations.

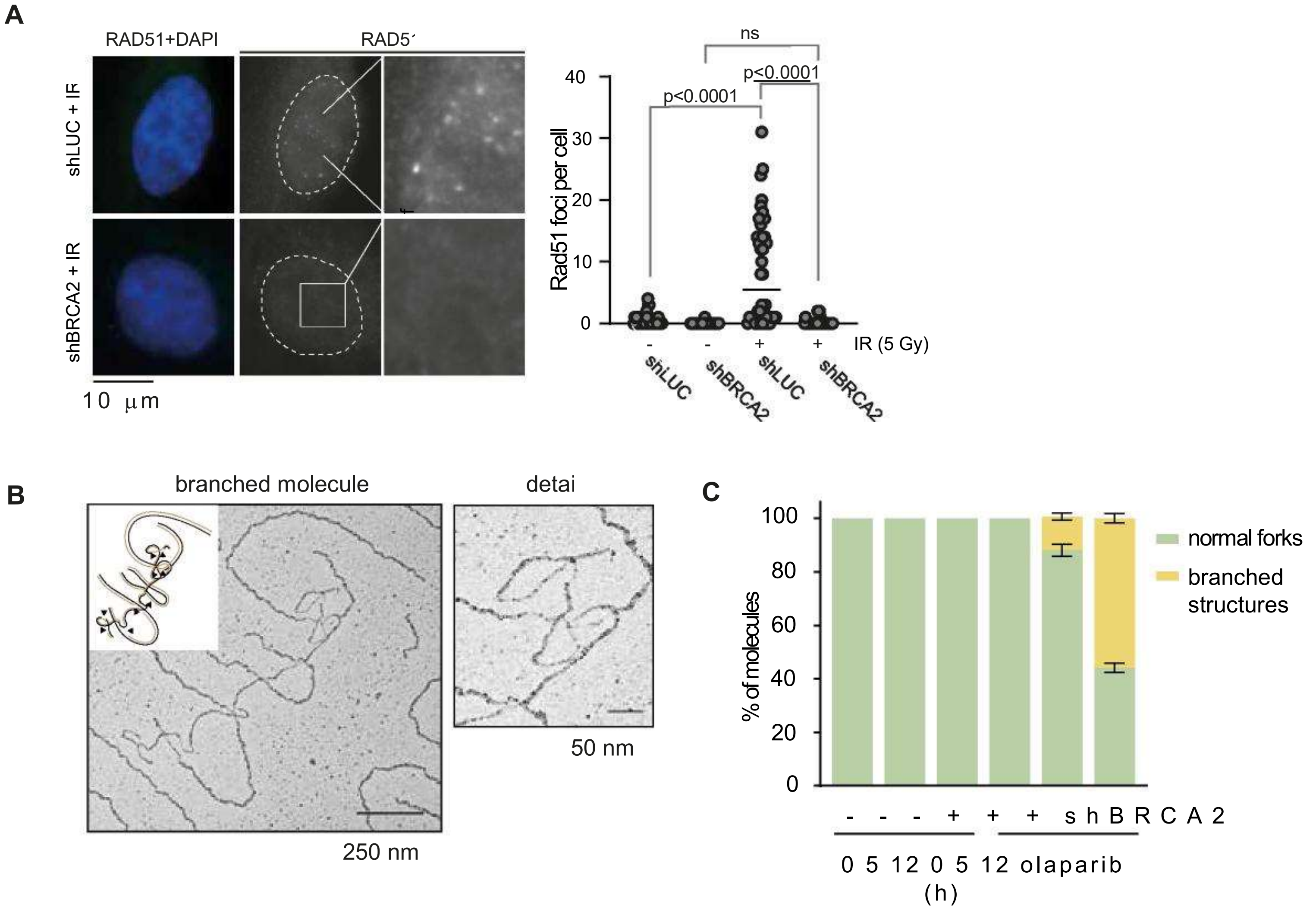

Supplementary Figure 2: BRCA2-depletion prevents RAD51 recruitment and induces branched DNA molecules. (A). RPE1 TP53-/- shBRCA2 were treated with doxycycline (dox) for 48 h, and irradiated with 5Gy if indicated and fixed 3h after irradiation. RAD51 foci were quantified by immunofluorescence microscopy. RAD51 foci per nucleus are plotted along with the mean of n = 38/37/42/36 cells per condition from one biologically independent experiment. Statistical analysis was done using a one-way ANOVA, with follow-up Sidak's tests. (B, C) RPE1 TP53-/-shBRCA2 cells were treated as for Figure 2B. Representative image of branched DNA molecule is shown along with a graphical interpretation (Panel B). Quantification of branched DNA molecules is indicated (panel C). Averages and standard deviations of 60 replication forks per condition are shown. Source data are provided with this paper.

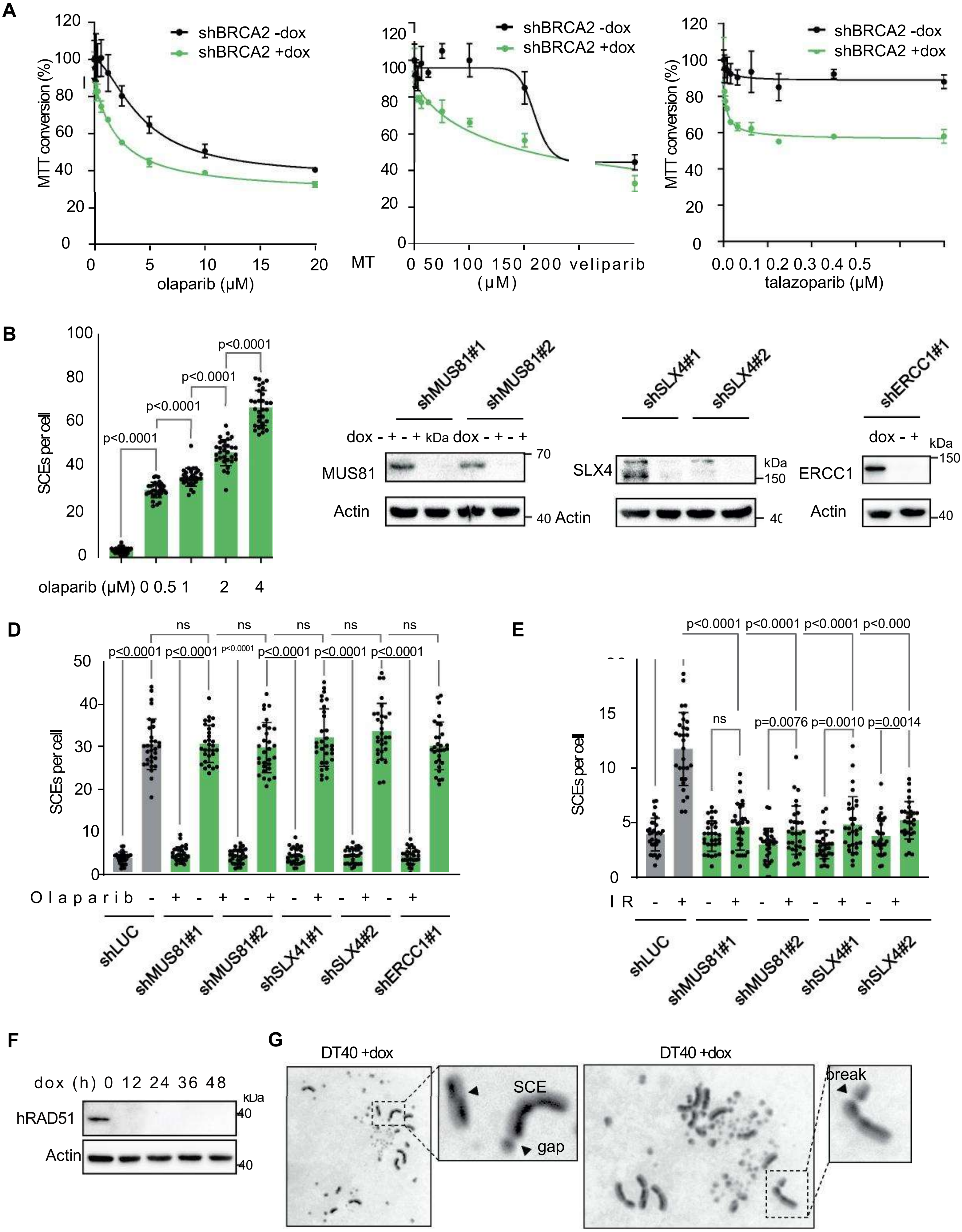
A
shBRCA2 -dox
shBRCA2 +dox
MTT conversion (%)
olaparib (μM)
MT
veliparib (μM)
talazoparib (μM)
B
SCEs per cell
p<0.0001
olaparib (μM) 0 0.5 1 2 4
shMUS81#1
shMUS81#2
shSLX4#1
shSLX4#2
shERCC1#1
dox
kDa
MUS81
Actin
SLX4
ERCC1
D
ns
Olaparib
shLUC
shMUS81#1
shMUS81#2
shSLX41#1
shSLX4#2
shERCC1#1
E
p=0.0076
p=0.0010
p=0.0014
IR
F
dox (h) 0 12 24 36 48
hRAD51
Actin
G
DT40 +dox
SCE
gap
break

Supplementary Figure 3: Olaparib-induced SCEs are dose-dependent and arise independently of canonical endonucleases.

(A) RPE1-*TP53*$^{-/-}$ shBRCA2 cells were pre-treated with doxycycline (dox) where indicated and subsequently treated with increasing doses of PARP inhibitors olaparib, veliparib and talazoparib for 3 days. MTT conversion was measured and used as a proxy for cell viability. Means and standard deviations of n=3 technical replicates from one biological experiment are shown. **(B)** RPE1 *TP53*$^{-/-}$ shBRCA2 cells were pre-treated with doxycycline (dox) and treated with increasing doses of olaparib for 48 hours. Means and standard deviations of 30 mitoses per condition are plotted. **(C)** RPE1 *TP53*$^{-/-}$ cells with indicated dox-inducible shRNAs were treated for 48 hours with doxycycline and immunoblotted for indicated proteins. **(D, E)** RPE1 *TP53*$^{-/-}$ cells with indicated dox-inducible shRNAs were treated with doxycycline for 48 hours, and subsequently treated with olaparib for 48 hours (panel D) or 2 Gy irradiation (panel E). SCEs were determined by differential BrdU incorporation. Means and standard deviations of 30 mitoses per condition (except n =31 for shLUC/olaparib in panel D) from one biologically independent experiment are plotted. Exact n values are indicated in the figure. **(F)** DT40 *RAD51*$^{-/-}$ cells harboring a dox-repressed hRad51 transgene were treated with doxycycline for indicated time periods, and immunoblotted for Rad51 and Actin. **(G)** DT40 *RAD51*$^{-/-}$ cells harboring a dox-repressed hRad51 transgene were treated with doxycycline for indicated time periods. Gaps and breaks are indicated with black arrows. Of note, DT40 cells have 11 macrochromosomes (included for analysis) and 67 microchromosomes (not included for analysis). Statistics in panels (B, D, E) were performed using unpaired two-tailed t-tests (ns: non-significant). Grey bars indicate HR-proficient conditions, green bars indicate HR-deficient conditions. Source data are provided with this paper.

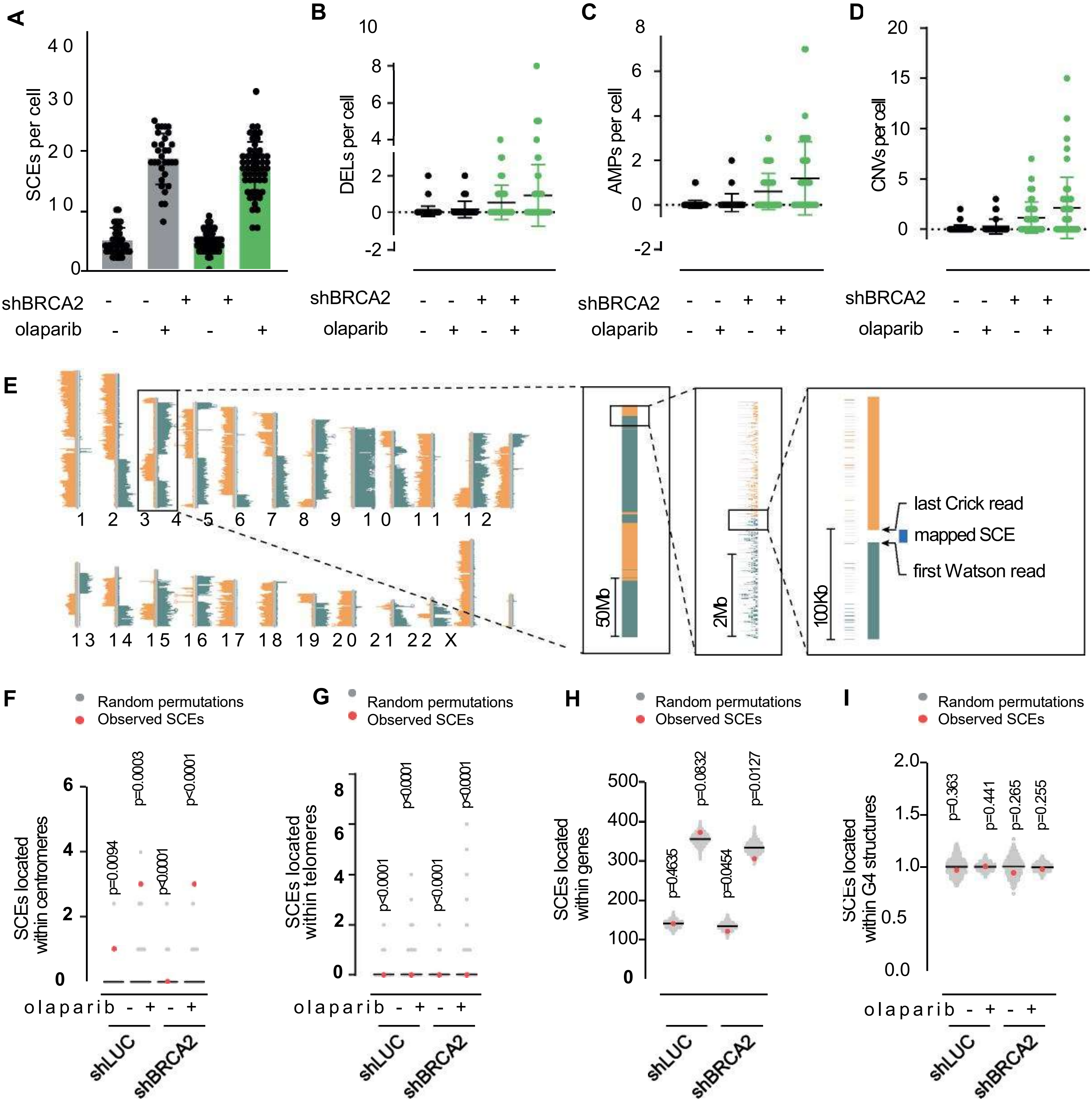

Supplementary Figure 4: Mapping of olaparib-induced SCEs in KBM-7 cells.

(A-D) KBM-7 cells harboring doxycycline-inducible shLUC or shBRCA2 were pre-treated with doxycycline and subsequently treated with olaparib if indicated. SCEs (panel B), deletions (DELs; panel C), amplifications (AMPs; panel C) and copy number variations (CNVs; panel D) were scored using StrandSeq of n=64 (shLUC/DMSO), n=31 (shLUC/OLA), n=50 (shBRCA2/DMSO) and n=52 (shBRCA2/OLA) libraries per condition. **(E)** Representative scheme displaying SCE mapping based on the gap between the last Crick read and the first Watson read. **(F-I)** Mapping of SCEs to centromeres (panel F), telomeres (panel G), gene bodies (panel H) and G4 structures (panel I) were computed. Statistical analysis was done using permutation tests (10,000 iterations). Observed SCEs and permutations are plotted. P values indicate deviation of the observed number of SCEs compared to the mean of all permutations. Grey bars indicate HR-proficient conditions, green bars indicate HR-deficient conditions.

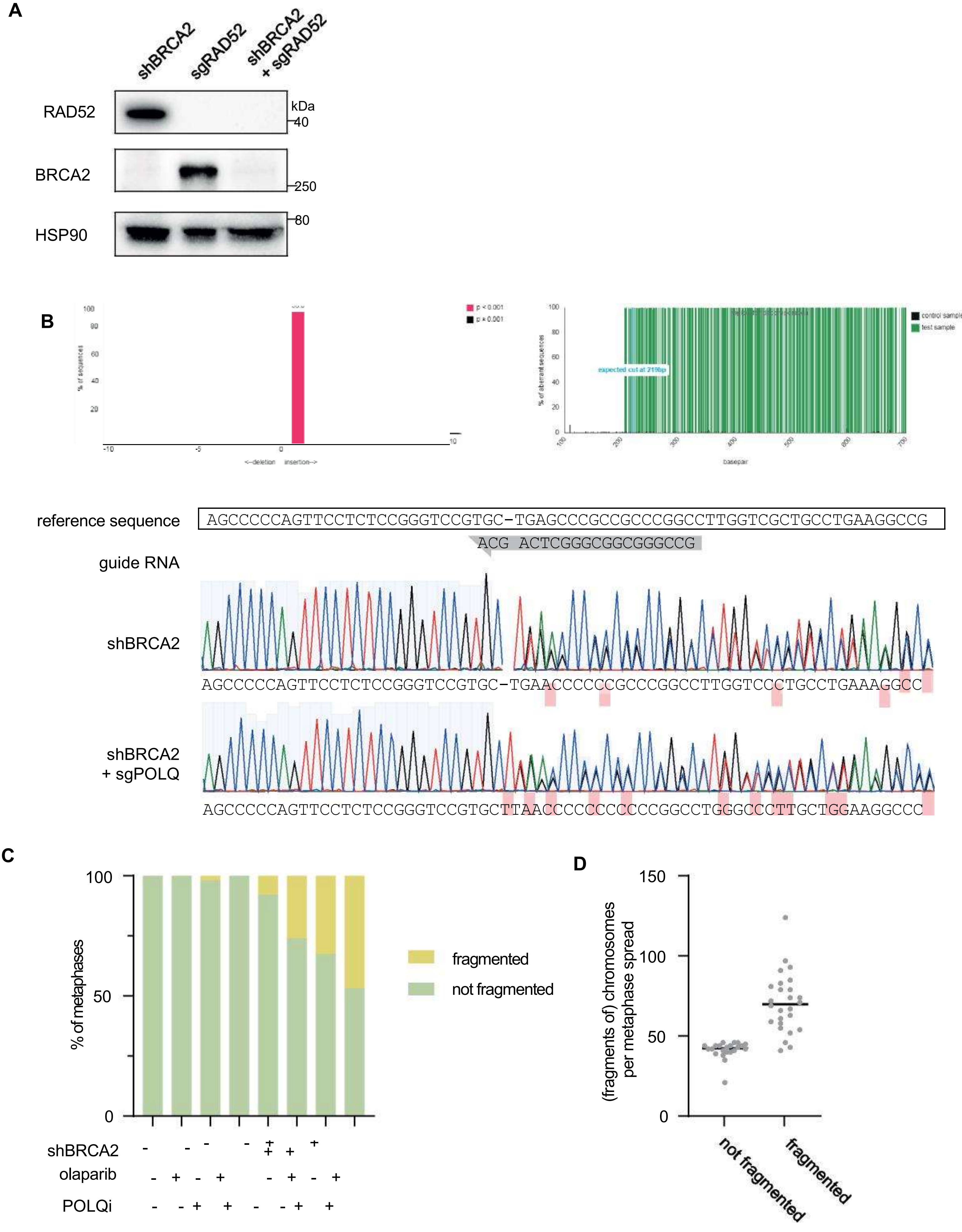
A
shBRCA2
sgRAD52
shBRCA2 + sgRAD52
RAD52
BRCA2
HSP90
kDa
40
250
80
B
p < 0.001
p ≥ 0.001
% of sequences
<--deletion
insertion-->
control sample
test sample
expected cut at 219bp
% of aberrant sequences
basepair
reference sequence
AGCCCCCAGTTCCTCTCCGGGTCCGTGC-TGAGCCCGCCGCCCGGCCTTGGTCGCTGCCTGAAGGCCG
guide RNA
ACG ACTCGGGCGGCGGGCCG
shBRCA2
AGCCCCCAGTTCCTCTCCGGGTCCGTGC-TGAACCCCCCGCCCGGCCTTGGTCCCTGCCTGAAAGGCC
shBRCA2 + sgPOLQ
AGCCCCCAGTTCCTCTCCGGGTCCGTGCTTAACCCCCCCCCCCGGCCTGGGCCCTTGCTGGAAGGCCC
C
% of metaphases
fragmented
not fragmented
shBRCA2
olaparib
POLQi
D
(fragments of) chromosomes per metaphase spread
not fragmented
fragmented

Supplementary Figure 5: Characterization of RAD52 and POLQ-deficient cell lines.

(A) RPE1 *TP53*$^{-/-}$ cells with dox-inducible shBRCA2 and CRISPR/Cas9-mediated RAD52 knockout were treated for 48 hours with doxycycline and immunoblotted for indicated proteins. Data are representative for two biologically independent experiments. **(B)** *POLQ* mutation in RPE1 *TP53*$^{-/-}$ shB RCA2 cells was assessed with TIDE and aligned to the reference genome, revealing a 1 bp insertion in exon 1. **(C)** RPE1 *TP53*$^{-/-}$ shBRCA2 cells were pre-treated with doxycycline (dox) and subsequently treated with olaparib or POLQi where indicated. Means and standard deviations of at least 50 mitoses per condition are plotted. **(D)** Numbers of chromosomes or chromosome fragments per metaphase spread were counted for RPE1 *TP53*$^{-/-}$shBRCA2 cells (n=48), that were pre-treated with doxycycline (dox) and subsequently treated with olaparib and POLQi. Source data are provided with this paper.

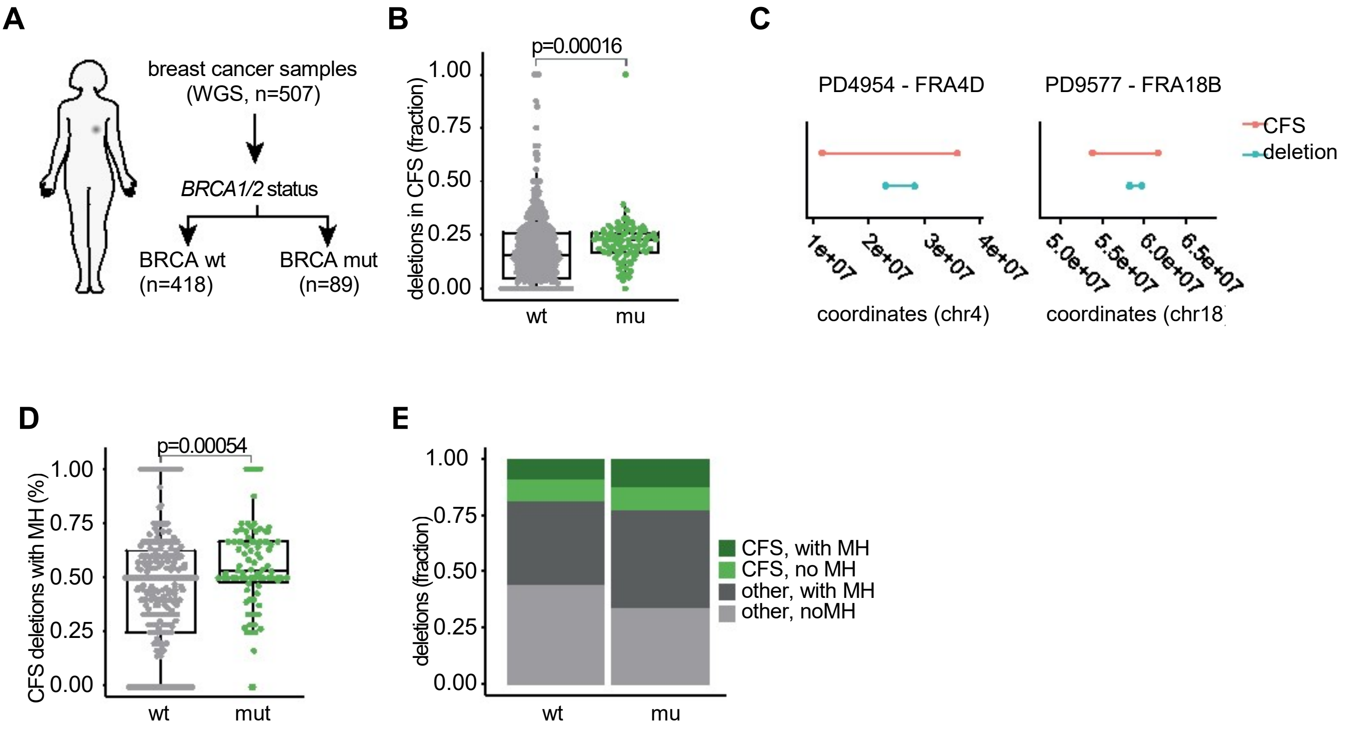

Supplementary Figure 6: Analysis of genomic deletions at common fragile sites. (A) Whole-genome sequence (WGS) data of n=507 breast cancers of the International Cancer Genome Consortium (IC GC) were analyzed, of which n=89 were *BRCA1/2* mutant. **(B)** Allelic deletions mapping to common fragile sites were plotted from n=507 breast cancers. Box plots depict the mean (center line), 25th and 7 5th percentiles (box boundaries), and the largest values no more than 1.5* the interquartile range (whiskers). Statistical analysis was done using an FDR-corrected two-sided Wilcox test. **(C)** Examples of all elic deletions mapping to common-fragile sites. **(D)** Allelic deletions mapping to common fragile sites and having microhomology (MH) (larger or equal to 2bp) at the breakpoints were plotted from n =507 breast cancers. Box plots depict the mean (center line), 25th and 75th percentiles (box boundaries), and the largest values no more than 1.5* the interquartile range (whiskers). Statistical analysis was 2 done using an FDR-corrected two-sided Wilcox test. **(E)** Analysis of deletions within CFS with or without MH as a percentage of total deletions were plotted.
