# Supplementary material for: Sister chromatid exchanges induced by perturbed replication can form independently of BRCA1, BRCA2 and RAD51
Source: Nat Commun. 2022 Nov 7;13:6722. doi: 10.1038/s41467-022-34519-8 (PMC9640580; doi:10.1038/s41467-022-34519-8)
Supplement: Supplementary file 3 — Description of Additional Supplementary Data [file 41467_2022_34519_MOESM3_ESM.pdf]

Description of Additional Supplementary Files

File Name: Supplementary Data 1
Description: Sister chromatid exchanges mapping to common fragile sites. Supplemental Dataset 1 corresponds to Figure 3C and Supplemental Figure S4. Genomic coordinates are indicated of fragile site, and the SCEs that map within these fragile sites, along with cell line and treatment information. WT refers BRCA2-proficient cells, DEF refers to BRCA2-deficient cells.

File Name: Supplementary Data 2
Description: Proteomics analysis of mitotic interactors of DNA ends. Supplemental Dataset 2 corresponds to Figure 5A/B. Xenopus leavis and Homo sapiens protein names are indicated.
