# Supplementary material for: Sister chromatid exchanges induced by perturbed replication can form independently of BRCA1, BRCA2 and RAD51
Source: Nat Commun. 2022 Nov 7;13:6722. doi: 10.1038/s41467-022-34519-8 (PMC9640580; doi:10.1038/s41467-022-34519-8)
Supplement: Supplementary file 6 — Reporting Summary [file 41467_2022_34519_MOESM6_ESM.pdf]

nature portfolio

Corresponding author(s): Marcel van Vugt; Peter Lansdorp

Last updated by author(s): Oct 10, 2022

Reporting Summary

Nature Portfolio wishes to improve the reproducibility of the work that we publish. This form provides structure for consistency and transparency in reporting. For further information on Nature Portfolio policies, see our Editorial Policies and the Editorial Policy Checklist.

Statistics

For all statistical analyses, confirm that the following items are present in the figure legend, table legend, main text, or Methods section.

n/a	Confirmed	
☐	☒	The exact sample size (*n*) for each experimental group/condition, given as a discrete number and unit of measurement
☐	☒	A statement on whether measurements were taken from distinct samples or whether the same sample was measured repeatedly
☐	☒	The statistical test(s) used AND whether they are one- or two-sided<br>*Only common tests should be described solely by name; describe more complex techniques in the Methods section.*
☒	☐	A description of all covariates tested
☒	☐	A description of any assumptions or corrections, such as tests of normality and adjustment for multiple comparisons
☐	☒	A full description of the statistical parameters including central tendency (e.g. means) or other basic estimates (e.g. regression coefficient) AND variation (e.g. standard deviation) or associated estimates of uncertainty (e.g. confidence intervals)
☐	☒	For null hypothesis testing, the test statistic (e.g. *F*, *t*, *r*) with confidence intervals, effect sizes, degrees of freedom and *P* value noted<br>*Give P values as exact values whenever suitable.*
☒	☐	For Bayesian analysis, information on the choice of priors and Markov chain Monte Carlo settings
☒	☐	For hierarchical and complex designs, identification of the appropriate level for tests and full reporting of outcomes
☒	☐	Estimates of effect sizes (e.g. Cohen's *d*, Pearson's *r*), indicating how they were calculated

Our web collection on statistics for biologists contains articles on many of the points above.

Software and code

Policy information about availability of computer code

Data collection	EM images were processed and analyzed using the MAPS software (FEI) and ImageJ software. Live cell imaging was analyzed using Nikon NIS-elements software. western blots were analyzed using Quantity One/ChemiDoc XRS software (Bio-Rad). fluoresence microscopy was analyzed using Las-af software (Leica, Wetzlar, Germany). Flow cytometry analysis was done with flowjo software (Becton Dickinson).
Data analysis	Indexed bam files were aligned to mouse (GRCm38) or human genomes (GRCh38) using Bowtie254. Different R-based packages were used for the detection and mapping of breakpoints: Aneufinder2 was used for libraries with arbitrary copy number profiles (KB2P3.4 and KB2P3.4R3), while HapSCElocatoR (https://github.com/daewoooo/HapSCElocatoR) was used for libraries derived from the haploid cell line KBM-7. HapSCElocatoR is implemented in the R package fastseg. A custom Perl script was used for the permutation model (https://github.com/Vityay/GenomePermute).

For manuscripts utilizing custom algorithms or software that are central to the research but not yet described in published literature, software must be made available to editors and reviewers. We strongly encourage code deposition in a community repository (e.g. GitHub). See the Nature Portfolio guidelines for submitting code & software for further information.

Data

Policy information about availability of data

All manuscripts must include a data availability statement. This statement should provide the following information, where applicable:

- Accession codes, unique identifiers, or web links for publicly available datasets
- A description of any restrictions on data availability
- For clinical datasets or third party data, please ensure that the statement adheres to our policy

Mass spec data are available via ProteomeXchange with identifier PXD028670. Strand-seq data are available via the European Nucleotide Archive (ENA) with identifier PRJEB47697.

Field-specific reporting

Please select the one below that is the best fit for your research. If you are not sure, read the appropriate sections before making your selection.

☒ Life sciences ☐ Behavioural & social sciences ☐ Ecological, evolutionary & environmental sciences

For a reference copy of the document with all sections, see nature.com/documents/nr-reporting-summary-flat.pdf

Life sciences study design

All studies must disclose on these points even when the disclosure is negative.

Sample size	Sample size was determined based on previous experience to obtain statistical significance and reproducibility. Usually a minimum of 20-30 chromosome spreads are analysed, yielding reliable results. For analysis of foci higher numbers were analysed to obtain reliable data.
Data exclusions	no data was exluded.
Replication	All findings were reliably reproduced in multiple experiments.
Randomization	Randomization was not relevant for this study.
Blinding	No blinding was used during data analyses, as the genotypes or treatments are distinguishable by visible effects.

Reporting for specific materials, systems and methods

We require information from authors about some types of materials, experimental systems and methods used in many studies. Here, indicate whether each material, system or method listed is relevant to your study. If you are not sure if a list item applies to your research, read the appropriate section before selecting a response.

Materials & experimental systems

n/a	Involved in the study	
☐	☒	Antibodies
☐	☒	Eukaryotic cell lines
☒	☐	Palaeontology and archaeology
☒	☐	Animals and other organisms
☒	☐	Human research participants
☒	☐	Clinical data
☒	☐	Dual use research of concern

Methods

n/a	Involved in the study	
☒	☐	ChIP-seq
☐	☒	Flow cytometry
☒	☐	MRI-based neuroimaging

Antibodies

Antibodies used	BRCA2 (Calbiochem, OP95, 1:1000), BRCA1 (Cell Signaling, 9010, 1:1000), RAD51 (GeneTex, gtx70230, 1:1000), PARP1 (Cell Signaling, 9532, 1:1000), RAD52 (Santa Cruz, sc-365341, 1:250), SLX4 (BTBD12; Novus Biologicals, NBP1-28680, 1:1000), MUS81 (Abcam, ab14387, 1:1000), ERCC1 (Cell Signaling, 3885, 1:1000), HSP90 (Santa Cruz, sc-1055, 1:1000), and beta-Actin (MP Biomedicals, 69100 1:10000). Horseradish peroxidase (HRP)-conjugated secondary antibodies (DAKO)
Validation	All used antibodies are recommended by the manufacturers for the detection of proteins of human origin. Validation involved analysis of expected kDa weight, and for most proteins, knock-out or shRNA was done, which indicated if bands dissappear upon genetic inactivation.

Eukaryotic cell lines

Policy information about cell lines

Cell line source(s)	hTERT-immortalized human retina epithelial RPE-1 cells and HEK293T cells were obtained from ATCC. KBM-7 cells were a kind gift from Thijn Brummelkamp (The Netherlands Cancer Institute, Amsterdam, The Netherlands). DT-40 cells were a kind gift from Shinuchi Takeda (Japan).The KB2P3.4 and KB2P3.4R3 cell lines were a kind gift from Jos Jonkers (The Netherlands Cancer Institute, Amsterdam, The Netherlands).
Authentication	RPE-1 and HEK293T were authenticated using STR profiling.
Mycoplasma contamination	all cell lines were tested negative for mycoplasma
Commonly misidentified lines (See ICLAC register)	NA

Flow Cytometry

Plots

Confirm that:

☐ The axis labels state the marker and fluorochrome used (e.g. CD4-FITC).

☐ The axis scales are clearly visible. Include numbers along axes only for bottom left plot of group (a 'group' is an analysis of identical markers).

☐ All plots are contour plots with outliers or pseudocolor plots.

☒ A numerical value for number of cells or percentage (with statistics) is provided.

Methodology

Sample preparation	cells were fixed in ice-cold ethanol (70%) for at least 16 h.
Instrument	LSR-II flow cytometer (Becton Dickinson, Franklin Lakes, NJ, USA).
Software	flowjo software (Becton Dickinson)
Cell population abundance	10.000 single cell events were measured. Single cells were identified by FL2 area versus FL2 width.
Gating strategy	cells were gated in FSC/SSC to exclude extremely small and large events. Single cells were identified by FL2 area versus FL2 width gating based on PI signal. this is a standard cell cycle analysis procedure.

☐ Tick this box to confirm that a figure exemplifying the gating strategy is provided in the Supplementary Information.
